# Posterior fossa syndrome

**DOI:** 10.1007/s00381-026-07512-5

**Published:** 2026-11-05

**Authors:** Sebastian M. Toescu, Kristian Aquilina, Paul Klimo

**Affiliations:** 1 Department of Neurosurgery, Great Ormond Street Hospital, London, UK; 2 Department of Surgery, St. Jude Children’s Research Hospital, Memphis, TN USA

**Keywords:** Posterior fossa syndrome, Cerebellar mutism syndrome, Medulloblastoma

## Abstract

Posterior fossa syndrome (PFS), also known as cerebellar mutism syndrome (CMS), is a distressing postoperative complication affecting children undergoing craniotomy for infratentorial tumors, most frequently medulloblastoma. It is characterized by a distinct latency period followed by mutism, profound emotional dysregulation, and a broad spectrum of cerebellar motor and cognitive deficits. Diffusion MRI and tractography have consistently shown that structural damage to the proximal efferent cerebellar pathway—specifically the dentato-rubro-thalamo-cortical tract within the superior cerebellar peduncle—is strongly implicated as the anatomical driver of PFS. Historical series favored the telovelar over the transvermian surgical approach, although recent prospective multicenter data demonstrate equivalent risks between the two techniques, shifting the neurosurgical focus toward meticulous surgical technique and minimizing damage to eloquent tissue around the fourth ventricle. Validated clinical scoring tools, such as the Posterior Fossa Syndrome Questionnaire, standardize clinical phenotyping and are making their way into clinical practice. Increasingly advanced risk prediction models allow for precise preoperative risk stratification based on objective imaging markers. Pharmacological interventions, including zolpidem and selective serotonin reuptake inhibitors, have shown limited efficacy in mitigating acute symptoms. Although near-universal clinical improvement occurs over time, long-term neurocognitive and motor sequelae remain highly disabling. Combining advanced neuroimaging, sophisticated risk modeling and meticulous microsurgical technique offers the best paradigm to shift the narrative of PFS from an expected complication to a preventable one.

## Introduction

Posterior fossa syndrome (PFS), often used interchangeably with the term cerebellar mutism syndrome (CMS), is one of the most distressing complications in pediatric neurosurgery. The syndrome is defined by a characteristic latency period preceding the onset of mutism (or at least speech disturbance) and emotional dysregulation, often accompanied by a broad spectrum of cerebellar motor and cognitive impairments. While the clinical features are most pronounced in the acute phase of the syndrome (first few weeks to months), improvement is universal, but the recovery trajectory may be prolonged, and affected children can be left with permanent neurocognitive sequelae in the chronic phase. Although similar clinical phenotypes have been documented following infectious [1], traumatic [2], or vascular [3] insults, the vast majority of cases occur as a postoperative complication of craniotomy and resection of infratentorial tumors in children and are most commonly seen in medulloblastoma.

Recent years have seen a dramatic interest in the field, as highlighted by a 2025 bibliometric analysis finding a sharp increase in the number of reference counts in the literature [4]. There is now a global concerted effort to understand the neurobiology and long-term consequences of this all-too-frequent complication, as well as potential ways to avoid it. This review article is intended to summarize the recent literature on the clinical and radiological features of PFS, etiology, prevention strategies, and outcomes.

## Nosology

The historical recognition of PFS has evolved through nearly a century of neurosurgical observations. Initial descriptions date back to Bailey et al. [5], who identified a delayed onset state of stupor and speech impairment 3 days after resection of a fourth ventricle ependymoma. By 1958, Daly and Love had broadened this clinical picture to include the cognitive and affective dimensions of the syndrome [6]. While subsequent surgical series utilized various descriptors such as “pseudobulbar palsy,” “emotional incontinence” [7], and “postoperative mutism” [8], it was not until Rekate’s landmark 1985 study [9] that the speech deficit was firmly codified as “muteness of cerebellar origin,” grounding the syndrome neuroanatomically within the posterior fossa.

In recent years, the nosology of the condition has continued to evolve. The original effort to standardize nomenclature came with a 2016 consensus statement [10] in which the syndrome was termed “post-operative pediatric cerebellar mutism syndrome.” A decade on, other groups continue to use the term “posterior fossa syndrome” (which is the preferred terminology in this Review), perhaps to acknowledge the broader constellation of neuropsychiatric and motor symptoms evident. Systematic, prospective assessment of a large cohort showed not only the hallmark clinical features of speech disturbance, irritability/emotional lability, and ataxia but also the prevalence of pyramidal weakness, dysphagia, ocular abnormalities, extrapyramidal movement disorders, and apraxia [11]. Notably, the cognitive and behavioral dimensions of PFS share significant overlap with the cerebellar cognitive affective syndrome (CCAS) described by Schmahmann [12], reflecting a broader failure of cerebellar efferent output to supratentorial cognitive and limbic networks.

### Assessment of severity

The lack of a unified grading system historically hindered the ability to compare outcomes across institutions. Early assessments were often binary, reducing a complex, heterogenous syndrome to a single dichotomised variable of mutism. The first rating scale [13], which was used to report outcomes in major pediatric oncology clinical trials, used duration of symptoms as a proxy for severity, thus limiting its value in the acute phase of the condition. The St. Jude Children's Research Hospital (SJCRH) group used their extensive experience treating children with PFS to devise diagnostic criteria for the condition [14] and later codified this into a comprehensive rating scale, the Posterior Fossa Syndrome Questionnaire (PFSQ) [15]. The PFSQ characterizes deficits in the three main domains of PFS, namely speech, motor, and behavioral. Each symptom within these domains is flagged as no/yes and includes dates of resolution to build in temporal data on symptomatology. In particular, speech/language changes, ataxia, and dysmetria were highly sensitive for PFS in this analysis. This rigorous approach to severity assessment yielded seminal results in their institutional series which dichotomised patients into PFS1 (those with mutism) vs PFS2 (those without mutism but other symptoms) [11]. Results from the Nordic group stratified patients into similar subgroups, but used the alternative terminology “postoperative speech impairment (POSI) 1 and 2.” Another rating scale has been proposed which includes oropharyngeal dysphagia, as well as ratings of duration and severity, which were strongly associated [16]. See Table 1 for a comparison of rating scales.

**Table 1:** Summary of rating scales used for posterior fossa syndrome

Reference	Focus	Domains scored	Key grading criteria	Disadvantages/limitations
Robertson (2006) [13]	Duration of mutism	Mutism Ataxia Hypotonia Irritability	**Mild:** < 1 week **Moderate:** 1–4 weeks **Severe:** > 4 weeks	Retrospective in nature Does not account for the *quality* of speech recovery
SJCRH PFSQ (2022) [15]	Comprehensive symptomatology	**Speech**: - Mutism - Language changes **Motor**: - Apraxia - Ataxia - Dysmetria - Tremor - Hemiparesis **Behavioral**: - Irritability - Excessive laughter	Multi-domain questionnaire (speech, motor, emotional)	**Complexity:** Can be time-consuming to administer in a busy clinical setting. Potential for **inter-observer variability** among different caregivers
SJCRH PFS1/2 (2021) [11]	Phenotyping Recovery	As above	**PFS1:** Mutism + emotional lability **PFS2:** Motor “stunning” without mutism	**Binary nature:** Does not grade the *severity* of the mutism itself (e.g., a child mute for 2 days is grouped with a child mute for 2 months)
Ricci (2022) [16]	Acute Diagnostic Grading	Mutism Emotional lability Hypotonia Oropharyngeal dysphagia	Duration and severity	**Narrow window:** Optimized for the acute phase (first month); may not capture the long-term neurocognitive “trajectory” accurately

## Imaging insights into neuroanatomy of PFS

**Fig. 1:**
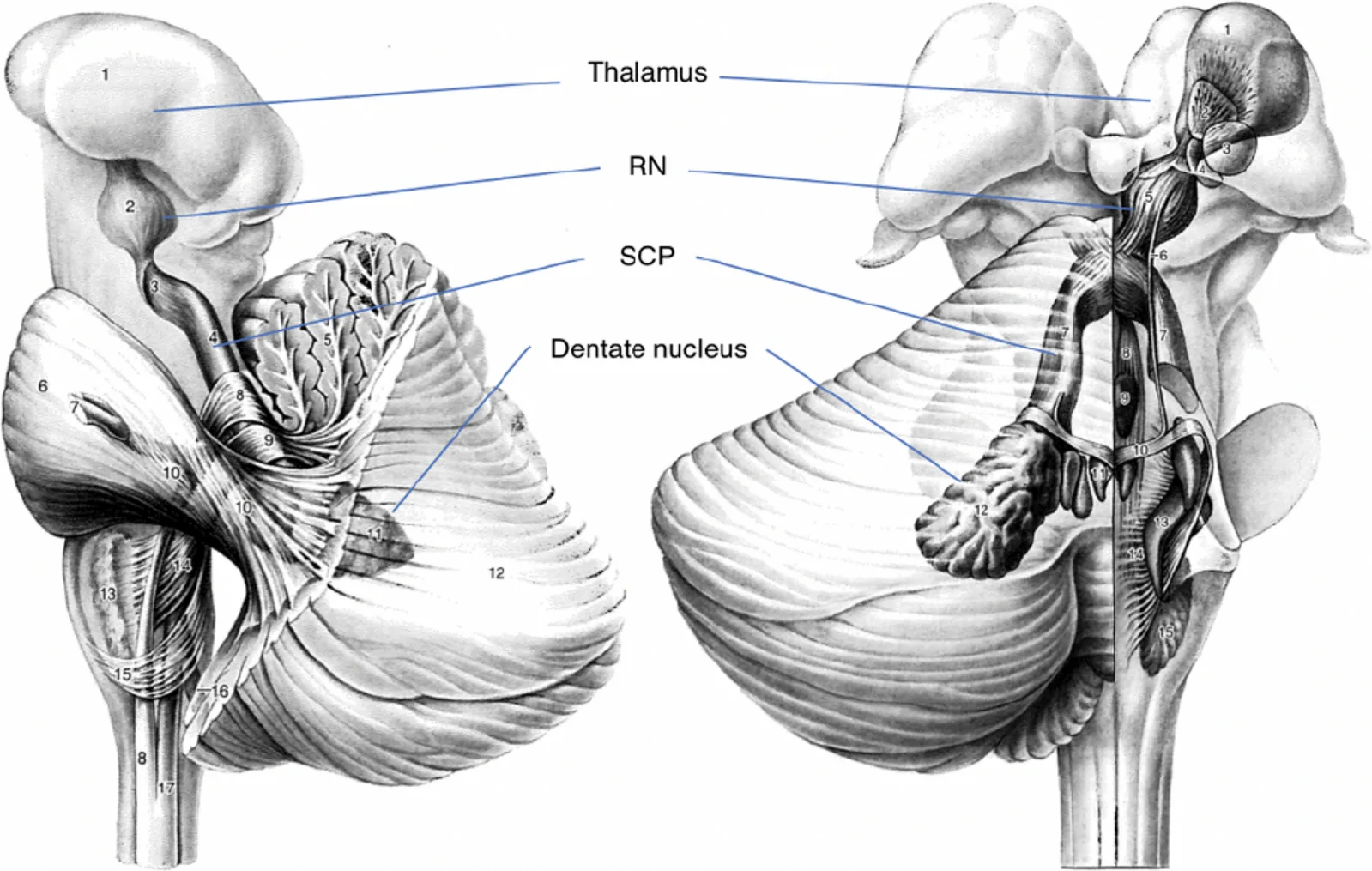
Elements of the proximal efference cerebellar outflow pathway, as viewed from the left lateral and posterior aspects. RN, red nucleus; SCP, superior cerebellar peduncle. Adapted by permission from Chapter 20 (Functional Systems: Cerebellum) in Springer Nature: Springer, The Human Central Nervous System by R. Nieuwenhuys, J. Voogd, and C. van Huijzen, © 2008

The shift from clinical description to etiological understanding of PFS has been driven by advances in neuroimaging which have begun to define the neural substrates of the condition. Damage to the proximal efferent cerebellar pathway (pECP; Fig. 1), comprising the portion of the dentato-rubro-thalamo-cortical (DRTC) tract as it ascends from the cerebellum to the midbrain via the superior cerebellar peduncle (SCP), has been strongly implicated in the development of PFS.

Diffusion MRI (dMRI), which uses specialized MRI sequences that are sensitive to the random motion of water molecules by diffusion, can be used to non-invasively delineate brain anatomy in vivo by inferring the local orientation of white matter fibers. Morris et al. used a diffusion-tensor tract-based analysis technique to demonstrate diffusion signal abnormalities in cerebellar outflow tracts in PFS [17], showing reduced fractional anisotropy (a measure of coherent fiber orientations) in the SCPs bilaterally in patients with PFS. This seminal finding led to a raft of studies showing abnormal signal in the pECP on voxel-based dMRI metrics [18–20].

Tractography, a technique in which local fiber orientations are iteratively averaged to construct streamlines approximating known white matter tracts, is a widely used tool in modern neurosurgical practice. Changes in SCP tractography in PFS were reported as early as 2012 [21–23]. These results have been progressively refined to show that tract changes are highly focal [24], and a greater degree of damage to the reconstructed DRTC tracts was associated with PFS severity [25]. Further anatomical insights have come from a voxel-based lesion symptom mapping study [26]—since validated in an independent cohort [27]—that showed lesions of the pECP and inferior vermis to be strongly associated with symptoms consistent with PFS. Pinto et al. demonstrated that patients with PFS had a higher incidence of SCP injury on immediate postoperative imaging, which translated into dystrophic mineralization of both dentate nuclei, suggestive of trans-synaptic retrograde degeneration [28]. The fastigial nucleus, the most medial of the cerebellar nuclei, is located in the roof of the 4th ventricle has also been implicated in the development of PFS [29]. In fact, Pinto et al. stated that “…preserving fastigial nuclear integrity is as important as avoiding direct SCP and dentate nuclear injury.”

The DRTC has wide-ranging connections to the cerebral cortex [30] which may go some way to explaining the symptoms of speech disturbance and emotional lability (functions typically ascribed to the forebrain) seen in PFS. dMRI can shine a light on microstructural changes in PFS, but its ability to elucidate functional changes or pathophysiological processes is limited. The missing link here is diaschisis [31, 32]—a sudden loss of function in a part of the brain distant to the index site of injury—which can be inferred based on perfusion MRI studies that have shown reduction in frontal lobe blood flow in patients with PFS [33, 34]. Taking a network neuroscience approach to the symptomatology of PFS, Ibrahim and colleagues demonstrated the intriguing finding that PFS results in an autism-like network disturbance [35]. Similarly, Chen et al. [36] used graph theory and connectomic analysis to decode symptomatologic dynamics in a deep-dive into an individual patient, providing a link between structural imaging and clinical manifestations in PFS.

## Incidence and risk factors

The earliest documented attempt at cerebellar tumor resection can be found in Harvey Cushing’s operative notes dating back to 1912 [37]; prior to Cushing’s historic advances, most cerebellar tumors in children were deemed inoperable. Surgery—specifically resection of a midline or 4th ventricular posterior fossa tumor in a child—is itself the biggest modifiable risk factor in the development of PFS. The Surgical Interest Group of the Posterior Fossa Society have recently published a consensus statement on surgical techniques to avoid PFS [38]. A number of recommendations were made, including the avoidance of tumor resection within a full posterior fossa, the avoidance of fixed brain retractors, and the avoidance of overusing bipolar cautery and ultrasonic aspirators near the floor of the fourth ventricle, SCPs, and deep cerebellar nuclei and preserving at all costs the integrity of the ependyma throughout the 4th ventricle.

Much attention has been given to the question of which method to enter and expose the 4th ventricle: transvermian versus telovelar. For most of the twentieth century, the transvermian approach was the favored technique. The telovelar approach, initially described by Matsushima and Rhoton [39, 40], exploits the cerebellomedullary fissure in order to divide the thin, membranous, non-eloquent tela choroidea (“telo”-) and inferior medullary velum (“velar”), thus mobilizing the cerebellar tonsil laterally and superiorly [41]. The cerebellar tonsils serve no significant neurologic function and therefore can be retracted essentially without consequence. In this manner, the fourth ventricle can be accessed without any transgression of neural tissue. Its clinical use was first described in a series of eleven pediatric fourth ventricle tumors [42], and since that time multiple series have demonstrated its utility in pediatric [43–48], adult [49], or mixed [50, 51] cohorts.

It was initially thought that the telovelar approach was less likely to lead to PFS than the transvermian approach [34, 52, 53]. More recently, several studies have demonstrated more equivocal findings [54–57]. The most robust evidence arises from the prospective observational study of 500 children by the Nordic group [58], which showed that the odds ratio of PFS was similar for the telovelar and transvermian approaches (OR 0.88, 95% CI 0.46–1.70; *p *= 0.18). This has led to a recommendation that the intradural surgical approach be tailored on a case-by-case basis, while at the same time aiming to transgress as little neural tissue as possible in the approach to the tumor.

The reported incidence of PFS varies between 8 and 50% in the literature at large, with a generally accepted aggregate rate of over 25% across multiple studies [56]. As PFS is rare after surgery for tumors in the cerebellar hemispheres, studies using fourth ventricular tumors or medulloblastomas as the denominator typically have higher PFS rates than studies which take into account all posterior fossa tumors. Most notably, some centers have reported significant reductions in PFS rate through modifications of surgical technique, such as avoidance of ultrasonic aspirator use and fixed retractor systems and a preference for the telovelar approach to the fourth ventricle [43, 59]. These efforts are to be applauded and emulated alongside the guidelines set out by the Posterior Fossa Society [38].

Nonmodifiable risk factors which have repeatedly been shown to increase the risk of PFS include medulloblastoma histology (in particular WNT and Group 3/4 molecular profiles [60]), brainstem or cerebellar peduncle infiltration [13], tumor size [21], younger age [61], and preoperative language deficits [11, 62]. The largest systematic review of risk factors associated with PFS [56] included fourth ventricle floor and SCP invasion, as well as left-handedness and vermian incision, although the results of the prospective Nordic study were not included in this meta-analysis. A protective effect was also found from the use of intraoperative imaging, either ultrasound [43, 54, 63] or intraoperative MRI [43], a finding which deserves robust prospective analysis in future studies. Salman et al. recently conducted another systematic review in which medulloblastoma histology, midline location, vermis incision, superior cerebellar peduncle involvement, and gross total tumor resection were identified as significant predictors of PFS [64].

Greater understanding of the preoperative non-modifiable risk factors has led to the development of tools to predict the risk of PFS in any given patient, based largely on imaging, demographics and likely tumor type (Table 2). An initial logistic regression-based model [65] had a high accuracy when tested on cohorts from two pediatric neurosurgery centers in the UK, but has not been externally validated. A second concise model, developed in Rotterdam [66], utilised a metric of brainstem displacement initially described by others [67] and improved upon the Liu model. Artificial neural networks were deployed in a model trained on data from three worldwide centers [68], which outperformed existing models [69] and is freely available as a web-based tool. The latest technological development in this area is the use of radiomic signatures which can identify microstructural features not visible to the human eye and conduct an objective textural analysis on voxel-level data in concert with a lesion symptom mapping approach [70, 71], but these models are yet to be tested.

**Table 2:** Comparison of clinical risk prediction models

Model	Study	Primary methodology	Key predictive variables	Clinical utility/strength
Nottingham	Liu et al. (2018) [65]	**Scoring system:** a weighted clinical-radiological score developed for routine practice	Includes variables such as brainstem invasion and tumor size/location	Designed for ease of use in a clinical setting to provide a quantitative risk percentage
Rotterdam	Dhaenens et al. (2020) [66]	**Validation and proposal:** evaluates existing MRI models to propose a refined Rotterdam pCMS model	Focuses on specific MRI-based anatomical markers and validated pre-operative features	Improved accuracy by validating and consolidating features from earlier predictive models
GOSH	Sidpra et al. (2022) [68]	**Machine learning:** utilizes an **artificial neural network (ANN)** to analyze complex data patterns	Leverages non-linear relationships between multiple imaging and clinical data points	Higher predictive performance compared to traditional linear scoring systems
Radiomics	Yang et al. (2023) [70] Chai et al. (2025) [71]	**High-dimensional data:** extracts “invisible” texture features from MRIs using advanced algorithms	Combines automated radiomic signatures with lesion-symptom mapping	Potential to identify microstructural risks not detectable by the human eye on standard imaging

## Treatment

The best treatment for PFS is prevention, which can be accomplished by meticulous surgical technique in high-volume centers which regularly manage children with posterior fossa tumors [38]. Once diagnosed, early engagement of ward-based therapy teams with patients with PFS is always indicated [72]. There are no protocolised medical treatments for PFS, although several medications are under investigation owing to propitious results in case reports and small series (Table 3). Zolpidem, a GABA-A agonist originally licensed as a hypnotic agent in 1992, was administered to a 4-year-old girl who developed PFS and mutism after surgery to resect a midline medulloblastoma. This had the paradoxical effect of increasing arousal and attentiveness and led to a return of speech within 14 days, with improvement back to her neurological baseline at 1 month. In a retrospectively reviewed case series of 19 patients with PFS, mutism scores improved by 64% in patients receiving zolpidem for 14 days [73]. These results lack control groups, and further research is needed in this area.

**Table 3:** Summary of medications used in the treatment of acute posterior fossa syndrome

Medication class	Example(s)	Proposed mechanism	Evidence quality	Key findings/limitations
GABA-A Agonists	**Zolpidem**	Paradoxical “awakening” effect; restores cerebral blood flow to the SMA and striatum by reversing diaschisis	**Case series/retrospective** (Lassaletta 2024; *n* = 19) [73]	**Finding:** 37% improved mutism scores within 48 h, 64% after 14 d of treatment **Limitation:** Sedation risk; results are often transient or inconsistent
Selective serotonin reuptake inhibitors (SSRIs)	**Fluoxetine, sertraline**	Modulates emotional lability and irritability via the limbic system; may improve neuroplasticity	**Matched cohort** (Xu/CHOP 2025; *n* = 24 pairs) [74]	**Finding:** Early initiation (within weeks) correlated with shorter mutism duration **Limitation:** No significant *overall* benefit compared to untreated patients in the total cohort
Dopamine agonists	**Bromocriptine**	Addresses “mesencephalic monoaminergic” damage; targets arousal and motor initiation centers	**Case reports** (Small *n*)	**Finding:** anecdotal reports of “spectacular” improvement in speech and motor initiation **Limitation:** potential for inducing psychosis or hallucinations in pediatric patients
Glucocorticoids	**Dexamethasone**	Reduction of postoperative edema in the deep cerebellar nuclei and brainstem	**Large prospective cohort** (Sarup 2025; *n* = 605) [78]	**Finding:** No evidence that higher doses or intraoperative use protect against speech impairment **Limitation:** High side-effect profile (hyperglycemia, weight gain) with no proven PFS-specific benefit
Benzodiazepines	**Midazolam, lorazepam**	Paradoxical restoration of speech, likely through GABAergic modulation of the cerebello-thalamo-cortical loop	**Case reports**	**Finding:** Some reports of “instantaneous” speech return following IV midazolam **Limitation:** Effect is typically very short-lived (minutes to hours)

Other groups have used antidepressants, with mixed results. In a large case series of 292 patients from Philadelphia, 24 children with PFS were treated with SSRIs [74]. Treatment did not decrease the duration of PFS symptoms compared to matched controls, although earlier initiation of fluoxetine was associated with a reduced duration of mutism. A small number of case reports exist of bromocriptine [75, 76] and midazolam [77] use in PFS, but a lack of control subjects makes it difficult to draw conclusions from this limited data. Post hoc analysis of data from the Nordic observational study have confirmed that perioperative glucocorticoid use does not reduce the risk of developing PFS [78].

## Outcomes

PFS can be a debilitating condition with long-term neurological and neurocognitive sequelae. The Nordic study provides the most robust prospective data on the early natural history of the condition [58]. Mutism typically occurs at a median of 1 day after surgery, and the median time to speech improvement was 16 days after onset. At 80 days after onset almost 90% of patients with mutism experienced improvement in speech. Residual neurological symptoms of ataxia and dysarthria are common [79]. Khan et al. report that no patient in their series with PFS (60/178) had a normal neurological examination at 12 months follow up and that almost half of patients with severe PFS were still unable to walk at that timepoint. From a neuropsychiatric perspective, the cardinal behavioral sign of irritability resolved at a median of around 1 month postoperatively [11].

Several reports have shown substantial longer-term neurocognitive morbidity in children with PFS, who are seen to have significantly lower full-scale IQ measures than healthy controls [80], and poorer cognition across multiple domains including processing speech, attention, executive function, and working memory [81]. While these results are confounded by the fact that most of the children with PFS in these cohorts are likely to have been treated with radiotherapy—which itself is an independent risk factor for neurocognitive deficits—analysis of the HIT-2000 trial shows that those with PFS had worse outcomes in almost all cognitive domains [82].

## Future directions

Our understanding of PFS has progressed dramatically in recent years, and there is a current climate of collaborative research across multiple centers worldwide, aided by regular interdisciplinary meetings of groups such as the Posterior Fossa Society. A better understanding of the clinical features of the syndrome has led to the development of multiple preoperative risk assessment tools and postoperative scoring scales which will increase the power of future research studies to detect treatment effects, and some new treatments are undergoing assessment by way of prospective trials. Further research is still required on the effect of centralisation of surgery; robust assessment of the benefit of intraoperative imaging, including real-time tractography of the pECP; and whether a change in focus on extent of resection in medulloblastoma will lead to a reduction in rates of PFS. By combining advanced imaging, AI-based risk prediction and refinement of surgical technique, the goal is to shift the narrative of PFS from an “expected complication” to one that is largely preventable.

## Data Availability

No datasets were generated or analysed during the current study.
